# Optimization of solution-processed oligothiophene:fullerene based organic solar cells by using solvent additives

**DOI:** 10.3762/bjnano.4.77

**Published:** 2013-10-24

**Authors:** Gisela L Schulz, Marta Urdanpilleta, Roland Fitzner, Eduard Brier, Elena Mena-Osteritz, Egon Reinold, Peter Bäuerle

**Affiliations:** 1 Institute of Organic Chemistry II and Advanced Materials, University of Ulm, Albert-Einstein-Allee 11, D-89081 Ulm, Germany; 2 Department of Applied Physics, University of the Basque Country (UPV/EHU), Plaza de Europa, 1, 20018 Donostia - San Sebastián, Spain

**Keywords:** active layer morphology, comparison vacuum-processed solar cells, maximum solubility, oligothiophene, solar cells, solution-processed bulk heterojunction, solvent additives

## Abstract

The optimization of solution-processed organic bulk-heterojunction solar cells with the acceptor-substituted quinquethiophene **DCV5T-Bu_4_** as donor in conjunction with PC_61_BM as acceptor is described. Power conversion efficiencies up to 3.0% and external quantum efficiencies up to 40% were obtained through the use of 1-chloronaphthalene as solvent additive in the fabrication of the photovoltaic devices. Furthermore, atomic force microscopy investigations of the photoactive layer gave insight into the distribution of donor and acceptor within the blend. The unique combination of solubility and thermal stability of **DCV5T-Bu_4_** also allows for fabrication of organic solar cells by vacuum deposition. Thus, we were able to perform a rare comparison of the device characteristics of the solution-processed **DCV5T-Bu_4_**:PC_61_BM solar cell with its vacuum-processed **DCV5T-Bu_4_**:C_60_ counterpart. Interestingly in this case, the efficiencies of the small-molecule organic solar cells prepared by using solution techniques are approaching those fabricated by using vacuum technology. This result is significant as vacuum-processed devices typically display much better performances in photovoltaic cells.

## Introduction

The demand for the development of new materials for applications in organic bulk-heterojunction solar cells (BHJSCs) has been growing over the last decade [1–3]. In response, the field has been expanding rapidly with the number of new compounds being produced at an increasingly faster rate [3–5]. The photoactive layer in BHJSCs is fabricated by simultaneous deposition of both, the electron donor (D) as a p-type and the electron acceptor (A) as n-type semiconducting material. The field can be divided based on the type of donor material; polymer or oligomer/dye molecules. Oligomers or, as they are sometimes referred to, “small” molecules, have the advantage of possessing a defined molecular structure that is monodisperse in nature and allows for purification and characterization, which leads to the derivation of valuable structure–property relationships. Problems with respect to reproducibility of solar cell results due to batch to batch variations of the synthetic organic materials, such as in the case of polymers, are of less significance. On the other hand, the preparation of structurally defined oligomers sometimes requires costly multi-step syntheses.

Diketopyrrolopyrroles [4], oligothiophenes [5], merocyanines [6], phthalocyanines [7], and squarine dyes [8–9] have all been investigated as promising donor materials in efficient BHJSCs. Power conversion efficiencies (PCEs) up to 6.9% have been reported for oligomers based on vacuum-processed [10] and 8.2% for solution-processed single junction devices [11]. Among these prominent classes of compounds, in particular oligothiophenes end-capped with electron-withdrawing cyano groups proved to have excellent performance in BHJSCs. Oligothiophenes of various lengths (from 3 to 7 thiophene units), which contain various alkyl side chains (methyl to octyl) with different substitution patterns, have been incorporated in photoactive layers of BHJSCs. When using vacuum deposition, the highest efficiency for single-junction solar cells to date has been reported to be 6.9% for dicyanovinyl (DCV)-capped quinquethiophene with methyl substituents on the central thiophene unit blended with C_60_ in a ratio of 2:1 [10]. In the case of solution-processed BHJSCs, a septithiophene derivative incorporating regioregular octyl chains, capped with DCV groups, and blended with [6,6]-phenyl-C_61_-butyric acid methyl ester (PC_61_BM), displayed a PCE of 3.7% when spin-coated from a chloroform solution [12]. This efficiency was further increased to 5.1% upon replacement of the terminal DCV acceptors units with octyl cyanoacetate termini [13]. A further improvement to 6.1% was obtained by the use of an alkylated septithiophene that bears terminal rhodanine acceptor groups [13]. Through combination of the rhodanine acceptor with a benzodithiophene core unit, an additional increase in PCE to 8.1% was achieved [14–15]. Simultaneously, a series of dithienosiloles flanked with two thiadiazolopyridine units were reported with efficiencies of up to 8.2% in combination with [6,6]-phenyl-C_71_-butyric acid methyl ester (PC_71_BM) [11,16–17].

We now report on the application of a DCV-capped quinquethiophene derivative, which contains four butyl chains along the oligomer backbone (**DCV5T-Bu_4_**), as the p-type semiconducting material in solution-processed BHJSCs. Due to its thermal stability as well as its solubility, this material has the unique advantage of being processable in both vacuum and solution. This allows for a direct comparison of the two deposition techniques and the resulting solar cell performances. There have been several reports describing the photovoltaic characteristics of vacuum-deposited **DCV5T-Bu_4_** [18–20], which in combination with C_60_ gave an efficiency of 3.4% in planar heterojunctions [18] and 3.5% in bulk heterojunctions [21]. Herein, the synthesis and characterization of the **DCV5T-Bu_4_** is described, as well as the photovoltaic performance of solution-processed BHJSCs. To date, there have been many reports of polymer-based solar cells, which have demonstrated significant increases in efficiencies with the use of solvent additives [22–28], however, there are only a handful of examples in which oligomer-based donors were used [15–17,29]. This work further investigates the effect of a solvent additive on active layer film formation and relates the findings to the solar cell performance [30].

## Experimental

**Materials and methods:** Tetrahydrofuran (THF, Merck) was dried under reflux over sodium/benzophenone (Merck) and distilled. Dimethylformamide (DMF, Merck) was first refluxed over P_4_O_10_ and distilled, then refluxed over BaO and distilled again. 1-Chloronaphthalene (CN, Aldrich) was distilled prior to use. All synthetic steps were carried out under argon atmosphere. Malononitrile and β-alanine were purchased from Merck and 2-isopropoxy-4,4,5,5-tetramethyl[1,3,2]dioxaborolane and thiophene were purchased from Aldrich. Diiodoterthiophene **1** [31], bisstannylterthiophene **5** [21], and 2-[(5-bromothien-2-yl)methylene]malononitrile (**6**) [32] were synthesized according to known literature procedures. NMR spectra were recorded on a Bruker AMX 500 (^1^H NMR: 500 MHz; ^13^C NMR: 125 MHz) or a Bruker Avance 400 (^1^H NMR: 400 MHz; ^13^C NMR: 100 MHz) at 298 K. Chemical shift values (δ) are given in ppm and were calibrated on residual non-deuterated solvent peaks (CDCl_3_: ^1^H NMR: 7.26 ppm, ^13^C NMR: 77.0 ppm; C_2_D_2_Cl_4_: ^1^H NMR: 6.00 ppm, ^13^C NMR: 74.0 ppm; CD_2_Cl_2_: ^1^H NMR: 5.32 ppm, ^13^C NMR: 53.5 ppm; THF-*d*_8_: ^1^H NMR: 3.58 ppm, ^13^C NMR: 67.7 ppm) as internal standard. EI and CI mass spectroscopy was performed on a Finnigan MAT SSQ-7000 or a Varian Saturn 2000 GCMS. MALDI-TOF spectra were recorded on a Bruker Daltonics Reflex III using dithranol or DCTB (trans-2[3-4-*tert*-butylphenyl]-2-methyl-2-propenylidene)-malononitrile) as matrices. UV–vis absorption spectroscopy was carried out on a Perkin Elmer Lambda 19 using Merck Uvasol grade solvents. The maximum solubility of **DCV5T-Bu_4_** was measured by using UV–vis absorption spectroscopy. After determination of the molar extinction coefficient, saturated solutions were made, stirred for 60 min at 60 °C then allowed to cool to room temperature. The saturated solution was then filtered and diluted for absorption spectroscopy, and the corresponding concentration could be determined. Cyclic voltammetry experiments were performed with a computer-controlled Autolab PGSTAT30 potentiostat and a three-electrode single-compartment cell with a platinum working electrode, a platinum wire counter electrode and an Ag/AgCl reference electrode. All potentials were internally referenced to the ferrocene/ferrocenyl couple (−5.1 eV). Melting points were determined using a Mettler Toledo DSC 823e and were not corrected. Elemental analyses were performed on an Elementar Vario EL. Plastic-sheets precoated with silica gel, Merck Si60 F254, were used for thin layer chromatography. Glass columns packed with Merck Silica 60, mesh 0.063–0.2 μm, were used for column chromatography. High performance liquid chromatography was performed on a Hitachi instrument equipped with a UV–vis detector L-7420, columns (Nucleosil 100-5 NO_2_ with a pore size of 100 Å) from Machery-Nagel using a dichlormethane/*n*-hexane mixture (40:60) as eluent. Surface images were recorded with the help of a Bruker Nanoscope V AFM at ambient temperature in tapping mode.

**Synthesis:** 3',3''',4',4'''-Tetrabutyl-2,2':5',2'':5'',2''':5''',2''''-quinquethiophene (**3**): Diiodoterthiophene **1** (2.77 g, 3.80 mmol) and 2-(thien-2-yl)-4,4,5,5-tetramethyl-[1,3,2]-dioxaborolane **2** (1.76 g, 8.36 mmol) were combined with a 2 M aqueous solution of potassium phosphate (12.5 mL, 25 mmol) in dimethoxyethane (60 mL). Tris(dibenzylideneaceton)dipalladium (41 mg, 0.04 mmol) and tri-*tert*-butylphosphine (16 mg, 0.08 mmol) were added to the reaction mixture under argon and it was refluxed for 24 h. After evaporation of the solvent, the crude product was purified by column chromatography on silica gel with petrol ether as eluent to yield pentamer **3** (1.70 g, 2.67 mmol, 70%) as an orange solid. Mp 72–73 °C; ^1^H NMR (CDCl_3_) δ 7.31–7.30 (m, 2H, ThH), 7.15–7.14 (m, 2H, ThH), 7.08–7.05 (m, 4H, ThH), 2.77–2.69 (m, 8H), 1.58–1.42 (m, 16H), 0.98-0.93 (m, 12H); ^13^C NMR (CDCl_3_) 140.19, 136.19, 135.94, 129.77, 127.39, 125.96, 125.90, 125.34, 32.97, 32.95, 28.01, 27.83, 23.09, 23.03, 13.93, 13.89. EIMS *m*/*z*: M^+^ 636.8 (calcd for C_36_H_44_S_5_: 636); Anal. calcd for C_36_H_44_S_5_: C, 67.87; H, 6.96; S, 25.17; found: C, 67.95; H, 6.36; S, 25.01.

3',3''',4',4'''-Tetrabutyl-2,2':5',2'':5'',2''':5''',2''''-quinquethiophene-5,5''''-dicarbaldehyde (**4**): To a solution of quinquethiophene **3** (1.50 g, 2.4 mmol) in dichloromethane (18 mL), a mixture of phosphoryl chloride in DMF (26 mL, 22.4 mmol) was added. The reaction was refluxed for 16 h and subsequently stirred for 2 h at room temperature. A saturated aqueous solution of sodium bicarbonate (200 mL) was added and the organic phase was extracted and dried over sodium sulphate. The crude material was purified by column chromatography on silica gel with dichloromethane as eluent to give dicarbaldehyde **4** (1.30 g, 1.88 mmol, 80%) as a dark red solid. Mp 89–90 °C; ^1^H NMR (CDCl_3_) 9.91 (s, 2H, CHO), 7.73 (d, *J* = 4.0 Hz, 2H, ThH), 7.28 (d, *J* = 4.0 Hz, 2H, ThH), 7.16 (s, 2H, ThH), 2.83–2.76 (m, 8H), 1.60–1.50 (m, 16H), 1.02–0.98 (m, 12H); ^13^C NMR (CDCl_3_) 182.53, 146.27, 142.62, 142.26, 140.84, 136.74, 135.94, 131.86, 129.01, 126.62, 126.15, 32.84, 32.56, 28.11, 27.92, 23.01, 13.84; MALDI-TOF *m*/*z*: M^+^ 692.3 (calcd for C_38_H_44_O_2_S_5_: 692). Anal. calcd for C_38_H_44_O_2_S_5_: C, 65.85; H, 6.40; S, 23.19; found: C, 65.95; H, 6.63; S, 22.93.

2,2'-((3',3''',4',4'''-Tetrabutyl-2,2':5',2'':5'',2''':5''',2''''-quinquethiophene-5,5''''-diyl)bis(methanylylidene))dimalononitrile (**DCV5T-Bu_4_**) by method (A): A suspension of dialdehyde **4** (0.80 g, 1.15 mmol), malononitrile (0.23 g, 3.45 mmol), and β-alanine (11 mg, 0.12 mmol) in THF/EtOH (1:3 mixture, 60 mL) was stirred for 20 h under reflux. The solvent was completely removed in vacuo and the resulting black solid was purified by column chromatography on silica gel with dichloromethane as eluent to yield **DCV5T-Bu_4_** (0.83 g, 1.05 mmol, 91%) as a dark violet to black solid.

2,2'-((3',3''',4',4'''-Tetrabutyl-2,2':5',2'':5'',2''':5''',2''''-quinquethiophene-5,5''''-diyl)bis(methanylylidene))dimalononitrile **DCV5T-Bu_4_** by method (B): A mixture of bisstannylterthiophene **5** (3.91 g, 4.90 mmol), 2-[(5-bromothien-2-yl)methylene]malononitrile **6** (2.46 g, 10.29 mmol) and tetrakis(triphenylphosphine)palladium(0) (283 mg, 0.245 mmol) was mixed in DMF (120 mL) and heated under argon at 80 °C for 72 h. After cooling, the resulting precipitate was filtered off and washed repeatedly with methanol and *n*-hexane. The DMF filtrate was then concentrated and stored at 7 °C and the resulting precipitate was filtered off and washed with methanol and *n*-hexane and combined with the previously isolated solid. Purification via column chromatography on silica gel was done using dichloromethane. After drying under vacuum **DCV5T-Bu_4_** (1.7g, 2.15 mmol, 46%) was obtained as a dark violet to black solid. The purity of quinquethiophene **DCV5T-Bu_4_** was confirmed by analytical high performance liquid chromatography (see Supporting Information File 1). Mp 204 °C (onset DSC). ^1^H NMR, ^13^C NMR, MALDI-TOF, and elemental analysis were all consistent with the previously reported values [20].

**Device fabrication:** Photovoltaic devices were made following a previously reported procedure [33], with a few exceptions. The active areas of the cells were 0.2 cm^2^. The spectral response was measured under monochromatic light from a 300 W Xenon lamp in combination with a monochromator (Oriel, Cornerstone 260), modulated with a mechanical chopper. The response was recorded as the voltage over a 220 Ω resistor, using a lock-in amplifier (Merlin 70104). A calibrated Si cell was used as reference.

## Results and Discussion

**Scheme 1:**
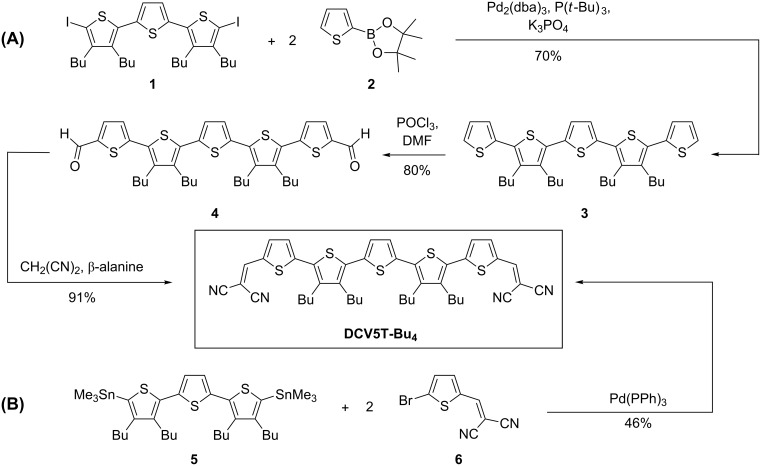
Alternative synthetic routes used to yield **DCV5T-Bu_4_**.

Two different synthetic strategies were employed to synthesize the DCV-capped quinquethiophene **DCV5T-Bu_4_** (Scheme 1). In the first approach (A) we started with the preparation of quinquethiophene **3** by Pd(0)-catalyzed Suzuki-type cross-coupling reaction of butyl-substituted diiodoterthiophene **1** and boronic ester **2** in 70% yield. For the sequential introduction of the terminal DCV acceptor groups, pentamer **3** was formylated in both α-positions under Vilsmeier–Haack conditions to yield dialdehyde **4**, which was subsequently converted into the target compound by a Knoevenagel condensation with malononitrile using β-alanine as catalyst. We recently developed the more versatile synthetic route (B), in which the already DCV-functionalized terminal thiophene **6** was coupled with bis-stannylated butyl-substituted terthiophene **5** in a two-fold Pd(0)-catalyzed Stille-type coupling reaction to obtain **DCV5T-Bu_4_** on the gram scale in 46% yield. After purification by column chromatography, the high purity and thermal stability of oligomer **DCV5T-Bu_4_** were confirmed by analytical high performance liquid chromatography (HPLC) and differential scanning calorimetry (DSC), respectively (see Supporting Information File 1).

**Figure 1:**
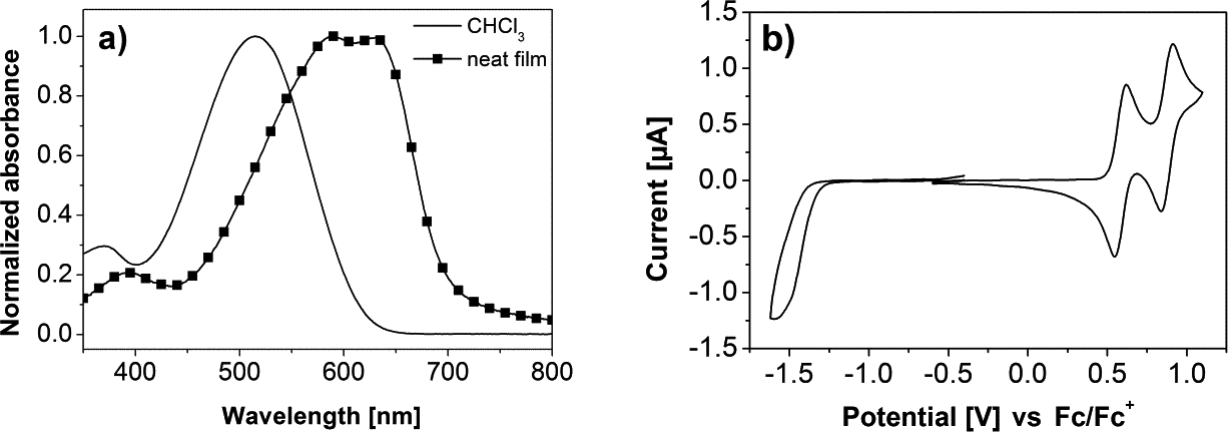
(a) Absorption spectrum of **DCV5T-Bu_4_** measured in chloroform and as thin film, spin-coated from chlorobenzene at 80˚C. (b) Cyclic voltammogram of **DCV5T-Bu_4_** in dichloromethane, TBAPF_6_ (0.1 M) measured versus the ferrocene/ferrocenyl (Fc/Fc^+^) redox couple.

The optical properties of **DCV5T-Bu_4_** were investigated by using UV–vis absorption spectroscopy and are displayed in Figure 1a and summarized in Table 1. In dilute chloroform solutions, absorption was observed between 400 and 600 nm, which is assigned to a π–π* transition. The maximum absorption was located at 515 nm, with a molar extinction coefficient of 62 300 L mol^−1^ cm^−1^ [20]. In comparison to the measurements performed in solution, thin films of neat **DCV5T-Bu_4_** showed a broader absorption profile that was shifted to the red with maxima at 590 and 630 nm. The onset of absorption was shifted for 87 nm, which reduces the band gap to 1.77 eV. The second maximum appearing at lower energy is attributed to the well-ordered packing of the **DCV5T-Bu_4_** molecules in the solid state.

**Table 1:** Optical, electrochemical, and maximum solubility data for **DCV5T-Bu_4_**, fullerene PC_61_BM, and PC_71_BM. Absorption spectra measured in CHCl_3_, thin films spin-coated from chlorobenzene at 80 °C and electrochemical measurements in CH_2_Cl_2_/TBAPF_6_ solutions (HOMO/LUMO vs Fc/Fc^+^_vac_ = −5.1 eV).

compound	λ_abs_ (nm) solution	ε (L mol^−1^ cm^−1^) solution	Δ*E*^opt^ (eV) solution	λ_abs_ (nm) film	Δ*E*^opt^ (eV) film	*E*^0^_ox1_ (V)	*E*^0^_ox2_ (V)

**DCV5T-Bu_4_**	515	62 300	2.03	590,630	1.77	0.58	0.87
PC_61_BM	329	40 100	3.08	—	—	—	—
PC_71_BM	470	22 100	—	—	—	—	—

compound	*E*^0^_red_ (V)	HOMO (eV)	LUMO (eV)	Δ*E*^CV^ (eV)	solubility CB (mg/mL)	solubility CN (mg/mL)	solubility ODCB (mg/mL)

**DCV5T-Bu_4_**	−1.50	−5.6	−3.7	1.87	3	6	3
PC_61_BM	—	−6.3 [34]	−4.0 [34]	—	31 [29]	31 [29]	—
PC_71_BM	—	−6.3	−4.1	—	—	—	164

**Figure 2:**
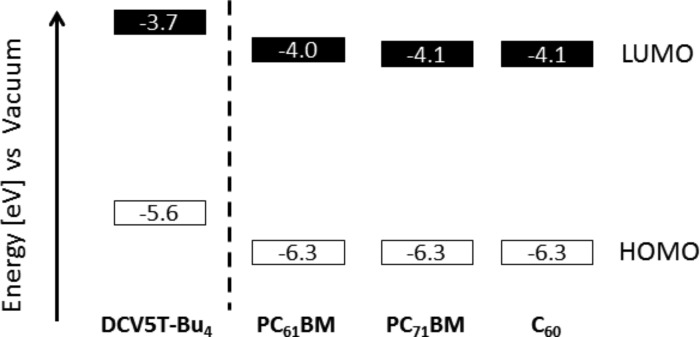
Diagram showing the HOMO and LUMO energy levels of **DCV5T-Bu_4_**, PCBM derivatives [34–35], and C_60_.

The electrochemical properties of **DCV5T-Bu_4_** were probed by using cyclic voltammetry, the results of which are plotted in Figure 1b and summarized in Table 1. Measurements were performed in dichloromethane solutions containing tetrabutylammonium hexafluorophosphate (TBAPF_6_) and referenced against the internal ferrocene/ferrocenyl (Fc/Fc^+^) redox couple. The first and second reversible oxidation of **DCV5T-Bu_4_** was observed at 0.58 and 0.87 V, respectively. Upon reduction of the molecule, an irreversible wave was observed at −1.50 V. The oxidation is attributed to the formation of stable radical cations and dications along the oligothiophene backbone, whereas the reduction corresponds to the more instable radical anions formed on the DCV groups. HOMO and LUMO energy levels were calculated to be −5.6 and −3.7 eV, respectively, from the onset of the first oxidation and reduction wave. The results are displayed in Figure 2 and compared to energy levels of three different electron-accepting fullerene derivatives used in the various experiments.

**Figure 3:**
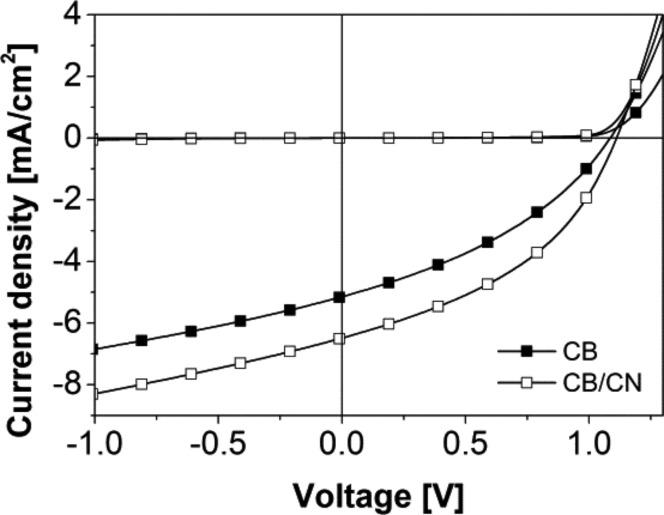
*J*–*V* curve of **DCV5T-Bu_4_**:PC_61_BM solution-processed solar cells made from 1:1 blends spin-coated from chlorobenzene solutions at 80 ˚C with (white squares) and without (black squares) the 1-chloronaphthalene additive.

Solar cell devices were fabricated by spin-coating the **DCV5T-Bu_4_**:PCBM blend from hot solutions at 80 °C on ITO|PEDOT:PSS-coated substrates, which were heated to 90 °C. Subsequently 1 nm LiF was deposited followed by 100 nm Al via thermal evaporation. *J*–*V* characteristics for blends of **DCV5T-Bu_4_**/PC_61_BM (1:1 wt. ratio) are displayed in Figure 3 and summarized in Table 2. When the active layer was deposited using only chlorobenzene (CB) as the solvent, a short-circuit current density (*J*_sc_) of 5.2 mA/cm^2^, an open circuit potential (*V*_oc_) of 1.09 V, a fill factor (FF) of 0.36, and a PCE of 2.1% were determined. As shown in Figure 2, the LUMO level of **DCV5T-Bu_4_** (−3.7 eV) was found to be about 0.3 eV higher in energy than that of PC_61_BM (−4.0 eV), which should be sufficient to enable efficient electron transfer at the donor–acceptor interface in the photoactive blend layer [36–37]. Moreover, the deep HOMO level, which is typically observed for acceptor-substituted oligothiophenes [32], implied that the *V*oc of the solar cell device should be quite high. Using the following empirical equation [37–38]:


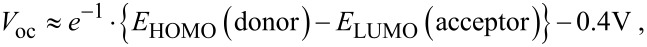


the expected *V*_oc_ can be calculated to be 1.2 V, which is 0.11 V higher than the measured value (1.09 V). Despite the very high V_oc_, a moderate PCE of 2.1% was obtained. The device made from CB displayed a relatively low fill factor (0.36), which is indicative of limited charge transport in the active layer. Furthermore, charge collection in the solar cell device may be limited by charge recombination, which is reflected in the high saturation value of 1.33 that was calculated by dividing the current density measured at −1 V by *J*_sc_ at short-circuit conditions (sat. = *J*(−1 V)/*J*_sc_(0 V)) [21].

**Table 2:** Photovoltaic parameters of solar cells fabricated using **DCV5T-Bu_4_**:PCBM from chlorobenzene, chloronaphthalene as additive, and spin-coated at 80 °C. Device structure: ITO|PEDOT:PSS|**DCV5T-Bu_4_**:PCBM (1:1)|LiF|Al.

donor:acceptor	solvent	*J*_sc_ (mA/cm^2^)	*V*_oc_ (V)	FF	PCE (%)	*J*(−1 V)/*J*_sc_(0 V)

**DCV5T-Bu_4_**:PC_61_BM	CB	5.2	1.09	0.36	2.1	1.33
**DCV5T-Bu_4_**:PC_61_BM	CB:CN (0.125%)	5.3	1.11	0.36	2.1	1.37
**DCV5T-Bu_4_**:PC_61_BM	CB:CN (0.25%)	5.9	1.10	0.39	2.6	1.29
**DCV5T-Bu_4_**:PC_61_BM	CB:CN (0.375%)	**6.5**	**1.11**	**0.41**	**3.0**	**1.28**
**DCV5T-Bu_4_**:PC_61_BM	CB:CN (0.50%)	5.9	1.10	0.41	2.7	1.28
**DCV5T-Bu_4_**:PC_61_BM	CB:CN (0.75%)	6.1	1.11	0.40	2.7	1.31

**DCV5T-Bu_4_**:PC_71_BM	ODCB	5.7	1.08	0.40	2.5	1.34

In order to investigate the effect of a solvent additive on the photovoltaic performance, a series of devices was made by varying the amount of 1-chloronaphthalene (CN) in CB from 0.125 to 0.75% wt./vol. All results are shown in Table 2 and the *J*–*V* curve for the best performing device (0.375% CN in CB) is compared to the device without solvent additive in Figure 3. Upon incorporation of 0.375% CN the *J*_sc_ is increased to 6.5 mA/cm^2^, the *V*_oc_ remains similar at 1.11 V, and the FF increased to 0.41 resulting in a significant increase in PCE to 3.0%. The main reason for this improvement is believed to be the increase in charge generation, which is reflected in the higher *J*_sc_ (6.5 vs 5.2 mA/cm^2^), and an improved charge transport and collection, as evidenced by the higher fill factor (0.41 vs 0.37) and lower saturation value (1.28 vs 1.33), respectively. The EQE spectra shown in Figure 5b (vide infra) demonstrate that the **DCV5T-Bu_4_**:PC_61_BM devices generate a photocurrent in the range of 400 to 700 nm and display maximum conversion at 580 nm. The EQE at 580 nm was measured to 40% and 36% for solar cells made with and without solvent additives, respectively. Further information regarding the solar cell performance dependence on the donor–acceptor ratio is summarized in Table S2 in Supporting Information File 1.

**Figure 4:**
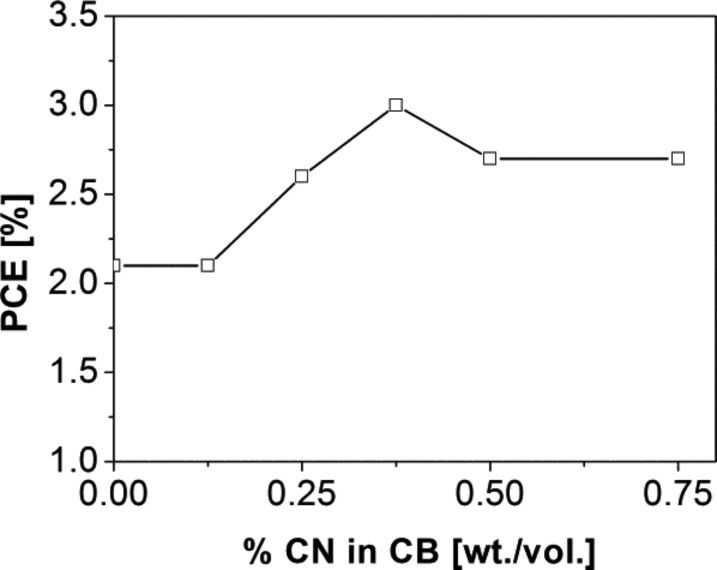
Power conversion efficiency of **DCV5T-Bu_4_**:PC_61_BM solution-processed solar cells as a function of CN content in CB.

Figure 4 demonstrates the dependence of the power conversion efficiency on the CN content in CB. From 0 to 0.375% CN, the PCE increased from 2.1% to a maximum value of 3.0%. Upon further increase of CN in CB to 0.50%, the device efficiency decreased to 2.7% and then leveled off. In order to investigate the solvent effect on the active layer formation, the maximum solubilities of **DCV5T-Bu_4_** and PC_61_BM were compared in both CB and CN (Table 1). PC_61_BM displays an equally high solubility in both CB and CN (31 mg/mL) [29], whereas **DCV5T-Bu_4_** is twice as soluble in CN as in CB (6 vs 3 mg/mL). We reason that this difference in solubility influences the active layer film morphology, which will be discussed in greater detail in a later section.

**Figure 5:**
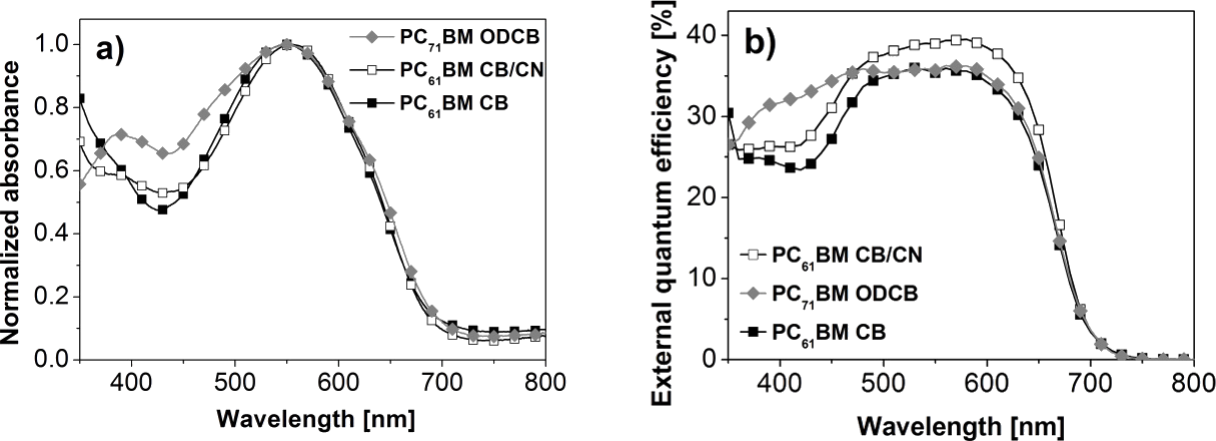
(a) Normalized absorption spectra of **DCV5T-Bu_4_**:PC_61_BM blends spin-coated from CB, CB:CN (0.375% wt./vol.) as well as **DCV5T-Bu_4_**:PC_71_BM blends spin-coated from ODCB. All films were spin-coated at 80 °C to accurately reproduce the active layer. (b) Spectral response plot of BHJ devices made using **DCV5T-Bu_4_** in combination with PC_61_BM (CB, CB:CN) or PC_71_BM (ODCB). Device structure: ITO|PEDOT:PSS|**DCV5T-Bu_4_**:PCBM|LiF|Al.

Further optimization of the **DCV5T-Bu_4_-**based active layer was done by investigating the effect of replacing the PC_61_BM electron acceptor with PC_71_BM. As PC_71_BM has a stronger absorption in the visible region of the solar spectrum than PC_61_BM, it was expected that the *J*_sc_ values of the corresponding solar cells that contain PC_71_BM would increase. The solar cells were fabricated in the exact same manner as those with PC_61_BM, except the solvent was changed to *o*-dichlorobenzene (ODCB). If one now compares the short-circuit current densities and efficiencies of the **DCV5T-Bu_4_**/PC_61_BM/CB device with the **DCV5T-Bu_4_**/PC_71_BM/ODCB device then an increase is observed (5.2 vs 5.7 mA/cm^2^ and 2.1 vs 2.5%, respectively). However an increase in the overall PCE for the optimized PC_61_BM (using a solvent additive) versus the optimized PC_71_BM active layer was not observed. In fact, the **DCV5T-Bu_4_**:PC_71_BM blends showed lower *J*_sc_ values (5.7 vs 6.5 mA/cm^2^), similar *V*_oc_, and FFs resulting in a lower PCE of 2.5% compared to the best **DCV5T-Bu_4_**:PC_61_BM device (3.0%). The stronger absorption of **DCV5T-Bu_4_**:PC_71_BM blends in the region from 400 to 500 nm is apparent in the normalized thin film absorption spectra shown in Figure 5a and in the photocurrent generated in the corresponding EQE spectrum (Figure 5b). However unfortunately, further attempts to improve the photovoltaic performance by using solvent additives in combination with PC_71_BM were unsuccessful (see Table S1 in Supporting Information File 1).

**Figure 6:**
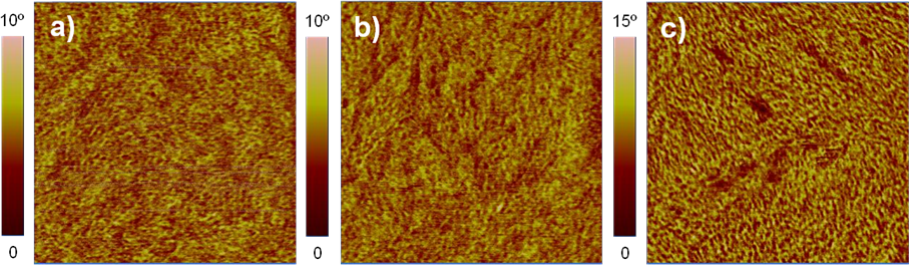
AFM phase images of samples spin-coated on ITO|PEDOT:PSS| with (a) **DCV5T-Bu_4_**:PC_61_BM from CB, (b) **DCV5T-Bu_4_**:PC_61_BM from CB:CN (0.375%), and (c) **DCV5T-Bu_4_**:PC_71_BM from ODCB. Image size: 1 × 1 μm.

The surface morphology of the D:A blend was investigated using atomic force microscopy (AFM). The samples were prepared in the same way as the photoactive layers for the solar cell devices; by spin-coating the **DCV5T-Bu_4_**:PCBM blends from hot solutions at 80 °C on ITO|PEDOT:PSS-coated substrates heated to 90 °C. Figure 6 depicts the phase images of **DCV5T-Bu_4_**:PC_61_BM and **DCV5T-Bu_4_**:PC_71_BM spin-coated from CB, CB with 0.375% CN, or ODCB, respectively. It is possible to assign the lighter regions (higher phase shift) to areas with mostly donor material (**DCV5T-Bu_4_**), whereas the darker regions (lower phase shift) contain mostly acceptor material (PCBM) [39]. The image shown in Figure 6a displays a relatively fine phase separation with domain sizes between 10–30 nm and a topography roughness averaged to be 0.4 ± 0.1 nm. Additionally, the film shown in Figure 6b, which was made with CN as solvent additive, displays similar domain sizes (10–30 nm) with a slightly lower topography roughness of 0.3 ± 0.1 nm. The histogram analysis taken over several images of different sizes gives a deeper insight into the corresponding D:A ratio (see Figure S3 in Supporting Information File 1). The surface of the photoactive layer deposited from the CB:CN mixture revealed a 14% higher amount of PC_61_BM than the film deposited from CB. The PCBM-rich regions are visible as dark depressions in the top left quadrant of the phase image shown in Figure 6b. Since the surface of the active layer under investigation contacts the cathode in the device, it would be reasonable to claim that the higher content of PCBM on the surface could lead to improved electron transport and collection in the photovoltaic device.

The observed changes in morphology between Figure 6a and 6b can be rationalized through the different solubility of **DCV5T-Bu_4_** in CN versus CB (6 vs 3 mg/mL). Since CN has a higher boiling point than CB (259 vs 132 °C), upon evaporation of CB during the final spin-coating stage, the CN content near the substrate increases. Considering the higher solubility of the oligothiophene in CN, we suggest that **DCV5T-Bu_4_**-richer domains are formed at the PEDOT:PSS interface. This hypothesis anticorrelates with the AFM results in which a PCBM-rich surface is found (vide supra), and both arguments explain the higher short-circuit current densities and fill factors observed in the solar cell devices made with the CN additive (see Table 2).

The **DCV5T-Bu_4_**:PC_71_BM blend depicted in Figure 6c shows large domains of PC_71_BM up to 100 nm in size (darker regions) and a topography roughness averaged to be 0.4 ± 0.1 nm. Thus, implementation of PC_71_BM led to large phase separation and consequently limited charge generation resulting in a reduction in short-circuit current densities (6.5 vs 5.7 mA/cm^2^) and PCEs (3.0 vs 2.5%) in the solar cell device. The non-ideal phase separation of **DCV5T-Bu_4_** and PC_71_BM spin-coated from ODCB can also be rationalized by using the relative maximum solublities of the donor and acceptor in the casting solvent. The oligothiophene donor displays a maximum solubility in of 3 mg/mL versus the PC_71_BM acceptor that shows a value of 164 mg/mL (see Table 1). We reason that it is this large difference in solubility of the electron donor and acceptor in ODCB that leads to a large phase separation and overall lower PCE (2.5 vs 3.0%) in the solar cell device containing PC_71_BM and PC_61_BM, respectively. This is in agreement with work done by Troshin et al., in which they correlated maximum solubilities of dozens of fullerene derivatives with maximum solar cell performances. In their study they proposed that novel donor polymers should be tested in organic solar cells with fullerene derivatives that have a similar solubility in the used solvent [40].

Acceptor-substituted oligothiophene **DCV5T-Bu_4_** possesses the unique characteristic of being processable both in vacuum and from solution, which allows for a rare comparison of the two device types (Table 3). The previously published vacuum-deposited active layer generates a higher *J*_sc_ (7.9 vs 6.5 mA/cm^2^), a lower *V*_oc_ (1.02 vs 1.11 V), and a similar FF (0.43 vs 0.41) compared to their solution-processed counterparts, which finally leads to an increase in the overall PCE (3.5 vs 3.0%) [21]. The lower open-circuit voltage found in the vacuum-processed device is attributed to the decreased LUMO energy of C_60_ (−4.1 eV) versus PC_61_BM (−4.0 eV) (Figure 2). The superior values for *J*_sc_ and FF of the vacuum-processed device can in part be explained by a better molecular packing in the photoactive layer. It is well known that during vacuum deposition the evaporation rate and substrate temperature can be precisely controlled and may be optimized to create highly ordered domains of donor and acceptor material [41]. These crystalline domains allow for higher exciton diffusion lengths [42] and thus higher charge generation, and improve charge transport to the electrodes. The better photocurrent saturation values for the vacuum-deposited cells, 1.17 versus 1.28 for the solution-processed devices, indicate reduced recombination, resulting in increased charge collection. The relatively modest difference in solar cell efficiency (3.0 vs 3.5%) for the two fabrication methods demonstrates the versatility of our **DCV5T-Bu_4_** material in contrast to, e.g., a merocyanine dye reported in literature (2.9 vs 4.9%) [43] or squarine dye (2.7 vs 4.1%) [44].

**Table 3:** Comparing vacuum [21] and solution-processed active layers of optimized solar cells fabricated from **DCV5T-Bu_4_**. Solution-processed device structure: ITO|PEDOT:PSS|**DCV5T-Bu_4_**:PC_61_BM|LiF|Al.

donor:acceptor	solvent	D:A ratio	*T*_soln/sub_ (°C)	J_sc_ (mA/cm^2^)	V_oc_ (V)	FF	PCE (%)	*J*(−1 V)/*J*_SC_(0 V)	EQE (%)

**DCV5T-Bu_4_**:PC_61_BM	CB:CN (0.375%)	1:1	80/90	6.5	1.11	0.41	3.0	1.28	40
**DCV5T-Bu_4_**:C_60_**^21^**	—	2:1	—/90	7.9	1.02	0.43	3.5	1.17	62

## Conclusion

We have demonstrated that the acceptor-substituted quinquethiophene **DCV5T-Bu_4_** can be applied in solution-processed bulk-heterojunction solar cells. Power conversion efficiencies were increased from 2.1% to 3.0% by using chloronaphthalene as a solvent additive. Atomic force microscopy experiments revealed that an excess of PC_61_BM was present on the surface of the photoactive layer when the film was made with the additive. This finding was then correlated to the increased charge generation (*J*_sc_), improved charge transport (*J*_sc_, FF), and increased charge collection (*J*(−1V)/*J*_sc_(0 V)) observed in the *J*–*V* curve of the photovoltaic cells. Furthermore, a rare direct comparison of solution- and vacuum-processed solar cells was possible. The efficiency of the optimized **DCV5T-Bu_4_**:PC_61_BM device at 3.0% is approaching the value of the vacuum-deposited **DCV5T-Bu_4_**:C_60_ device, which has been previously reported to be 3.5%.

## Supporting Information

File 1 Further measurement data.
