# Supplementary material for: Optimization of solution-processed oligothiophene:fullerene based organic solar cells by using solvent additives
Source: Beilstein J Nanotechnol. 2013 Oct 24;4:680–9. doi: 10.3762/bjnano.4.77 (PMC3817626; doi:10.3762/bjnano.4.77)
Supplement: File 1 — Further measurement data. [file Beilstein_J_Nanotechnol-04-680-s001.pdf]

Supporting Information

for

Optimization of solution-processed oligothiophene:fullerene based organic solar cells by using solvent additives

Gisela L. Schulz*[1], Marta Urdanpilleta[2], Roland Fitzner[1], Eduard Brier[1], Elena Mena-Osteritz[1], Egon Reinold[1] and Peter Bäuerle*[1,§]

Address: [1]Institute of Organic Chemistry II and Advanced Materials, University of Ulm, Albert-Einstein-Allee 11, D-89081 Ulm, Germany and [2]Department of Applied Physics, University of the Basque Country (UPV/EHU), Plaza de Europa, 1, 20018 Donostia - San Sebastián, Spain

Email: Gisela L. Schulz* - gisela.schulz@uni-ulm.de;

Peter Bäuerle* - peter.baeuerle@uni-ulm.de

* Corresponding author

[§]Fax: (+49) 731-50-22840

Further measurement data

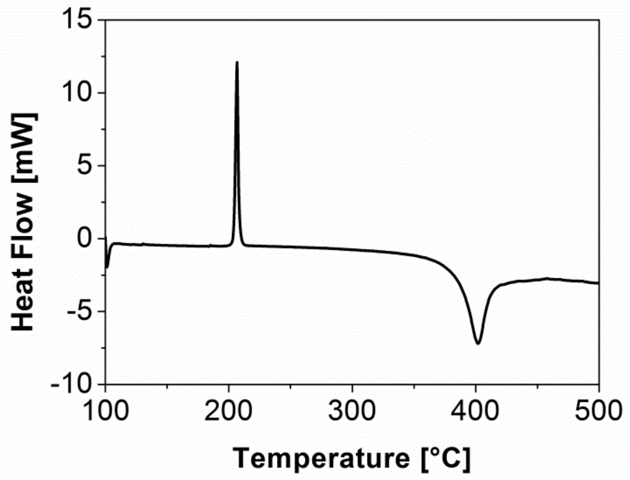

Figure S1: DSC trace of **DCV5T-Bu$_4$** after purification by column chromatography.

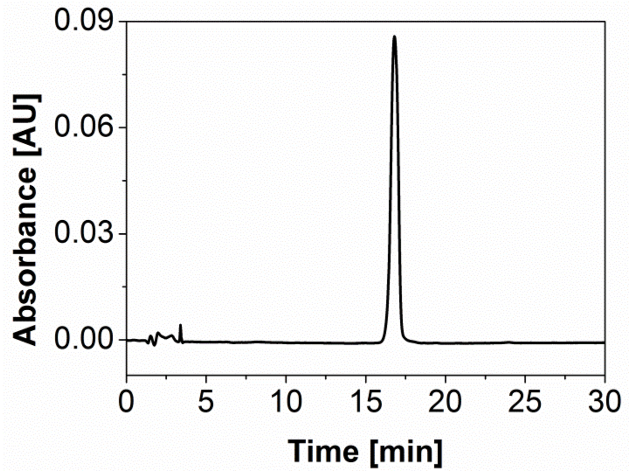

Figure S2: HPLC trace of **DCV5T-Bu$_4$** after purification by column chromatography. Detection wavelength 500 nm.

Table S1: Photovoltaic parameters of solar cells fabricated using **DCV5T-Bu$_4$** from CB or ODCB, using CN as additive and spin coated at 80 °C. Device structure: ITO|PEDOT:PSS|**DCV5T-Bu$_4$**:PCBM (1:1)|LiF|Al.

**Donor:Acceptor**	**Solvent**	**Jsc (mA/cm^2)**	**Voc (V)**	**FF**	**PCE (%)**
**DCV5T-Bu$_4$**:PC$_{71}$BM	CB	5.2	1.08	0.35	2.0
**DCV5T-Bu$_4$**:PC$_{71}$BM	CB:CN (0.375%)	5.6	1.09	0.39	2.4
**DCV5T-Bu$_4$**:PC$_{71}$BM	ODCB	5.7	1.08	0.40	2.5
**DCV5T-Bu$_4$**:PC$_{71}$BM	ODCB:CN (0.375%)	5.7	1.09	0.40	2.5

Table S2: Photovoltaic parameters of solar cells fabricated using **DCV5T-Bu$_4$** from CB or CB/CN with different D:A ratios and spin coated at 80 °C. Device structure: ITO|PEDOT:PSS|**DCV5T-Bu$_4$**:P$_{61}$CBM|LiF|Al.

**Donor:Acceptor**	**Donor:Acceptor ratio**	**Solvent**	**Jsc (mA/cm^2)**	**Voc (V)**	**FF**	**PCE (%)**
**DCV5T-Bu$_4$**:PC$_{61}$BM	1:2	CB	3.8	1.09	0.34	1.4
**DCV5T-Bu$_4$**:PC$_{61}$BM	1:1	CB	5.2	1.09	0.36	2.1
**DCV5T-Bu$_4$**:PC$_{61}$BM	3:2	CB	4.6	1.10	0.34	1.7
**DCV5T-Bu$_4$**:PC$_{61}$BM	1:1	CB:CN (0.5%)	6.0	1.10	0.41	2.7
**DCV5T-Bu$_4$**:PC$_{61}$BM	3:2	CB:CN (0.5%)	6.1	1.11	0.41	2.8
**DCV5T-Bu$_4$**:PC$_{61}$BM	1:0.8	CB:CN (0.375%)	6.0	1.11	0.42	2.8
**DCV5T-Bu$_4$**:PC$_{61}$BM	2:1	CB:CN (0.375%)	5.2	1.12	0.41	2.4

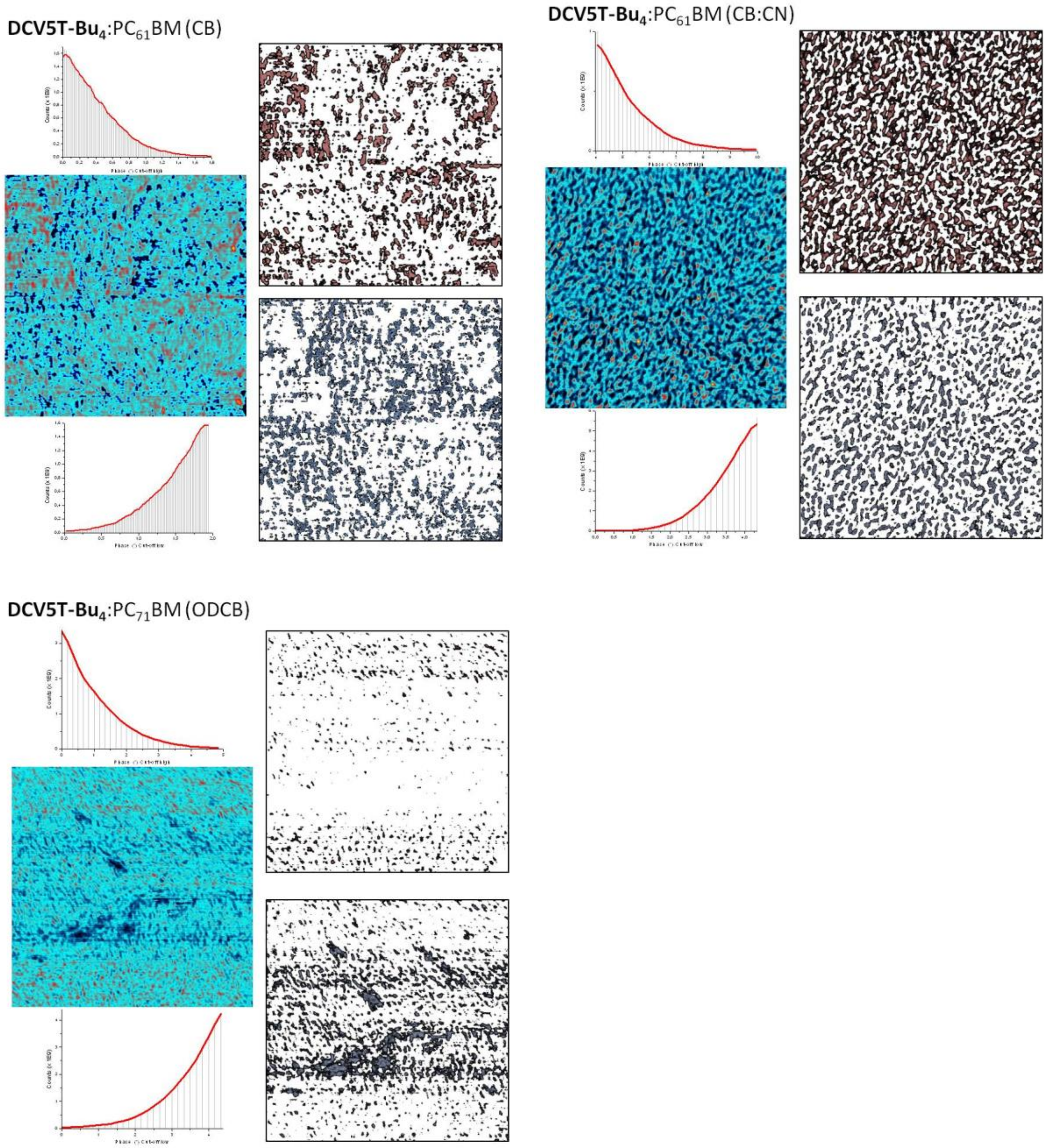

Figure S3: Representative phase images of the three blend materials (left) and separated histogram phases corresponding to the high (upper image) or low (bottom image) phase shift (Δ = 2–3°), related to the more-ordered phases of the two active materials.
